# Identification and gene expression profiling of toll genes in the potato psyllid (*Bactericera cockerelli*) in response to ‘*Ca*ndidatus liberibacter solanacearum’

**DOI:** 10.1007/s11033-026-12777-9

**Published:** 2026-09-22

**Authors:** Md Tafim Hossain Hrithik, Julien Levy, Taylor Hubbs, Cecilia Tamborindeguy

**Affiliations:** 1 Department of Entomology, Texas A&M University, TAMU 2475, College Station, TX 77843 USA; 2 Department of Horticultural Sciences, Texas A&M University, College Station, TAMU, TX 2133, 77843 USA

**Keywords:** Toll pathway, *Candidatus* Liberibacter solanacearum, *Bactericera cockerelli*, Gene expression

## Abstract

**Background:**

The Toll signaling pathway is a key component of insect innate immunity, yet its role in hemipteran vectors and their interactions with plant pathogens remains poorly understood. The potato psyllid (*Bactericera cockerelli*), an important agricultural pest in the USA, transmits two haplotypes of *‘Candidatus* Liberibacter solanacearum*’* (LsoA and LsoB) that cause severe diseases in solanaceous crops.

**Methods and Results:**

We characterized Toll pathway components in the *B. cockerelli* genome and examined their transcriptional responses during pathogen acquisition. Six Toll receptors and ten pathway-associated genes were identified in the psyllid genome. Gene expression was evaluated using RT-qPCR following exposure to the entomopathogenic fungus *Beauveria bassiana* (Bb-490) and infection with Lso haplotypes. The transcription of five Toll receptors was significantly induced in response to *B. bassiana*, indicating their potential role in antifungal defense. In contrast, Lso infection elicited haplotype-specific transcriptional responses: two Toll receptors (Toll like-1 and Toll-7) were up-regulated during LsoB acquisition, whereas only Toll-7 responded to LsoA. In addition, Spätzle-3 was induced by LsoB, while Spätzle was induced by LsoA. Persistent Lso infection did not significantly alter the transcriptional expression of Toll receptors.

**Conclusions:**

These findings provide new insights into immune regulation in *B. cockerelli* and highlight the potential of targeting Toll-mediated responses and fungal biocontrol strategies for sustainable disease management.

**Supplementary Information:**

The online version contains supplementary material available at https://doi.org/10.1007/s11033-026-12777-9.

## Introduction

*Bactericera cockerelli* Šulc (Hemiptera: Triozidae), known as the potato psyllid or tomato psyllid, is a serious economically-damaging pest of solanaceous crops, including potato and tomato [1]. It is indigenous to the southwestern United States and northern Mexico, but today can be found in the Western half of the United States, parts of Canada, Mexico, Central America, Ecuador, Colombia, New Zealand, and parts of Australia [2]. Potato psyllids feed from the vascular system, causing leaf chlorosis and decreasing the crop’s yield [3]. More critically, the potato psyllid is a vector of the pathogenic bacterium ‘*Candidatus* Liberibacter solanacearum’ (Lso) [4].

Lso is a phloem-limited, Gram-negative, and unculturable plant pathogen from the class of α-proteobacteria. Lso is transmitted by psyllids in a circulative and propagative manner [5, 6]. In the United States, LsoA and LsoB, two Lso haplotypes, cause severe diseases such as zebra chip in potatoes. Zebra chip is a highly destructive disease significantly decreasing potato yields and resulting in up to 85% losses in severely affected fields [7]. Currently, the prevention of Lso-related diseases relies on pesticide applications to manage the psyllid populations, as no economically viable genetic resistance has been discovered in the affected crops. Nevertheless, this strategy has limitations, and innovative management methods to block pathogen transmission are urgently needed.

Understanding the immune responses elicited in psyllids in response to Lso could pave the way to develop transmission disruption strategies. Insect immunity has been investigated in various species, with a particular focus on dipteran and lepidopteran insects [8, 9]. Insects lack adaptive immunity, but they possess a robust innate immune system [8] which is pivotal against pathogens and invading microorganisms [8, 10–13]. The insect innate immune system consists of humoral and cellular immune responses. Humoral responses rely on the activation of immune signaling pathways such as Toll, immune deficiency (IMD), and Janus kinase/signal transducers and activators of transcription (JAK/STAT) [14–17]. The activation of these pathways leads to different immune responses, including the production of antimicrobial peptides (AMPs) [15, 18, 19]. However, many hemipteran insects such as psyllids and aphids exhibit either a partial or completely absent IMD pathway [20–24]. In this context, the Toll pathway has been identified as potentially involved in the psyllid immune response (or lack of response) to liberibacter [25]. This study investigated the involvement of the Toll signaling pathway in the potato psyllid response to Lso. While Toll receptors can be identified in different species based on sequence similarity, the identification of the elicited antimicrobial peptides remains challenging. Here, we first analyzed the potato psyllid genome to identify genes involved in the Toll pathway. Then, we evaluated the expression of the identified candidate receptors following fungal challenge, which is known to induce the Toll pathway across insect species, as a way to identify which gene could be involved in the response to pathogens. Finally, we analyzed the expression of the Toll genes in response to the persistent infection by LsoA and LsoB in nymphs, male and female adults from infected colonies; these insects likely encountered the bacteria during their nymphal stages because Lso is not vertically transmitted. We also assessed their expression during the early stages of infection when key differences in the acquisition of LsoA and LsoB exist. This research marks the beginning of understanding the molecular mechanisms and potential role of the Toll pathway in the potato psyllid. This knowledge contributes to our understanding of immunity in psyllids and can help develop better strategies to control this important pest and/or the pathogens it transmits.

## Materials and methods

### Identification and bioinformatic analysis of toll receptor and other toll pathway-related genes

Putative Toll receptors and other genes involved in the Toll pathway were identified from the unpublished psyllid genome available in our laboratory ) based on similarities with previously characterized Toll pathway genes from other insect species. Initially, well-characterized genes involved in the Toll pathway from model organisms such as *Drosophila melanogaster*, *Acyrthosiphon pisum*, and *Diaphorina citri* were retrieved from the NCBI database and used as reference sequences. These sequences were used to perform tBLASTn searches against the *B. cockerell*i genome database using an E-value cutoff of 1 × 10^− 20^. We complemented this approach by performing BLASTn searches using our annotated transcriptome [24]. We only selected candidates encoding complete proteins based on similarities to other species’ genes. Candidate Toll pathway genes were further validated based on the presence of conserved protein domains using Prosite (https://prosite.expasy.org/*)* and InterPro (https://www.ebi.ac.uk/interpro/). SignalP 6.0 (https://services.healthtech.dtu.dk/services/SignalP-6.0*)* was used to detect N-terminal signal peptides [26] for both *B*. *cockerelli* and *D. citri*.

Multiple sequence alignments of Toll protein sequences from *B. cockerelli* and representative insect species were performed using the ClustalW algorithm implemented in MEGA6 with the default parameters. Phylogenetic relationships were inferred using the Maximum Likelihood method based on the Jones-Taylor-Thornton (JTT).

### Insect rearing and plant maintenance

All experiments used tomato plants (Moneymaker; Victory Seed Company, Molalla, OR). Lso-free, LsoA- and LsoB-infected psyllid colonies were maintained separately on tomato plants in insect cages (24 × 13.5 × 13.5 cm, BioQuip, Compton, CA). All plants and colonies were maintained at 24 ± 2 °C and under a photoperiod of 16: 8 h (L: D). To obtain Lso-infected plants for acquisition experiments, 4-week-old plants were infested with three adults from the LsoA- or LsoB-infected colonies caged in an organza bag for one week. Four weeks after insect removal, the plants were evaluated for infection by PCR as described in Tang and Tamborindeguy [27] using the LsoF/OI2 primers and the Lso haplotype in the plants was confirmed using the Lso SSR-1 primers [28].

### RNA extraction, cDNA preparation, and qPCR

For the expression analyses, five pools of 10 nymphs, adult females, or males were collected from the LsoA- and LsoB-infected and Lso-free psyllid colonies. For the time-dependent analyses following different Lso acquisition access periods (AAP), Lso-free female psyllids were transferred to LsoA- and LsoB-infected plants for 2, 3, 5, 7, and 14 days. The Lso-free female psyllids were used as a control. Ten pools of 10 females were created for each AAP; five pools were used for gene expression analysis and five for Lso quantification (described below). RNA from the pools was extracted using TRIzol reagent (Invitrogen, Carlsbad, CA, USA) according to the manufacturer’s instructions. The RNA was treated with Turbo DNase (Ambion, Invitrogen, Waltham, MA, USA) and used to synthesize complementary DNA (cDNA) using the Verso cDNA Synthesis kit (Thermo, Waltham, MA, USA) and anchored-Oligo (dT) primers according to the manual. The cDNA was used as a template for quantitative PCR (qPCR) analysis using gene-specific primers (Table S1) and the Power SYBR Green Master Mix (Applied Biosystems, Waltham, MA, USA). The reaction mixture (10 µL) contained 5 µL of Power SYBR Green PCR Master Mix, 2 µL of cDNA template, 0.5 µL of forward and reverse primers (250 nM-final concentration), and 2 µL of deionized distilled water. The RT-qPCR program was 95 °C for 2 min, followed by 40 cycles at 95 °C for 5 s and 60 °C for 30 s. RT-qPCR assays were performed using a QuantStudio™ 6 Flex Real-Time PCR System (Applied Biosystems). The elongation factor-1a and ribosomal protein subunit 18 were used as reference genes [29]. All reactions were performed in triplicate. PCR efficiency of each reaction was determined using LinRegPCR software [30]. All reactions were performed with five biological replicates. The expression of the candidate genes was calculated using the minus ∆Ct (-∆Ct) method.

### Fungal bioassay and gene expression analysis

The fungal pathogen *Beauveria bassiana-490* (Bb-490) was cultured in potato dextrose agar (Difco, Fisher Scientific, USA) for 14 days. The third instar (L3) nymph of the potato psyllid was used for this experiment, in which the leaf-dipping method was used for bioassays. Briefly, conidial suspensions of the Bb-490 were prepared by scraping the surface of the fungal culture and placing it into a falcon conical tube containing autoclaved Triton X-100 (0.01%) solution. To separate the conidial clumps, the suspension was stirred for 2–5 min with a Vortex mixer. Conidial concentrations in the suspensions were measured using a hemocytometer (Marienfeld-Superior, Paul Marienfeld GmbH and Co. KG, Lauda-KoÈnigshofen, Germany) under a 40× microscope.

A piece of tomato leaf (approximately 4 cm^2^) was dipped into a fungal suspension (10^7^ conidia/mL) for 10 min. After soaking, the leaf was dried for 5 min under laminar airflow in a hood. After drying, the treated leaf was placed in a Petri dish and 20 randomly chosen nymphs were released on the leaf. After 48 h, the treated leaf was replaced with a fresh one. For the control treatment, a Triton X-100 (0.01%) solution (without fungus) was used. The plates were placed at 25 °C with a relative humidity of 70%. Data was collected daily for up to 6 days. Each treatment was replicated three times with 20 nymphs per replication.

The expression of the Toll genes was analyzed in L3 nymphs after a 48-hour immune challenge with Bb-490 and control treatment. RNA extraction, cDNA synthesis, and qPCR analyses were performed as described above using gene-specific primers.

### DNA extraction and quantification of Lso

For the time-dependent analysis of Lso bacterial copy numbers following different acquisition access periods (AAPs), Lso-free adult female psyllids were transferred to LsoA- or LsoB-infected plants for 2, 3, 5, 7, or 14 days. Following each AAP, DNA was extracted using the DNeasy Blood & Tissue Kit (Qiagen, Hilden, Germany).

Lso titers in five pools of ten adult female psyllids per AAP were quantified using Lso 16 S rDNA-specific primers [31], while psyllid 28 S rDNA primers [32] were used as an internal control. The reaction mixture (10 µL) contained 5 µL of Power SYBR Green PCR Master Mix, 2 µL of DNA template, 0.5 µL of forward and reverse primers (250 nM final concentration), and 2 µL of deionized distilled water. The qPCR program consisted of an initial denaturation at 95 °C for 2 min, followed by 40 cycles of 95 °C for 5 s and 60 °C for 30 s, and reactions were run on a QuantStudio™ 6 Flex Real-Time PCR System (Applied Biosystems, Foster City, CA). To standardize the amount of Lso in psyllid, data are reported as delta Ct = (Ct of Lso gene) – (Ct of psyllid 28 S gene). The average delta Ct value was used to quantify the levels of Lso. A standard curve was prepared for the quantification of Lso in the psyllid using a plasmid containing the Lso 16 S rDNA target. For the standard curve, 10-fold serial dilutions of the plasmid were performed. The procedure for standard curve preparation followed published methods [33]. The Lso copy number in each sample was estimated by comparing the delta Ct values of each sample to the standard curve.

### Statistical analysis

All data analyses were carried out with JMP Version 16 (SAS Institute Inc., Cary, NC, USA) and GraphPad Prism 9 Software (GraphPad Software, San Diego, CA, USA). Survival was analyzed using Kaplan-Meier survival curves and log-rank tests. Gene expression results upon Lso infection were analyzed with one-way ANOVA with Tukey’s *post hoc* test for multiple pairwise comparisons among treatments. To evaluate the effects of developmental stage, treatment, and their interaction on gene expression, a two-way ANOVA was performed, followed by Tukey’s test to determine significant differences among groups. The effects of the fungus on the expression of Toll genes were determined with Student’s t-tests. For the analysis of LsoA and LsoB copy number in the psyllids, the data were log_10_-transformed and analyzed using a two-way ANOVA with Tukey’s *post hoc* test. All values are presented as mean ± SE.

## Results

### Identification of toll receptors and other toll pathway-related genes

**Fig. 1:**
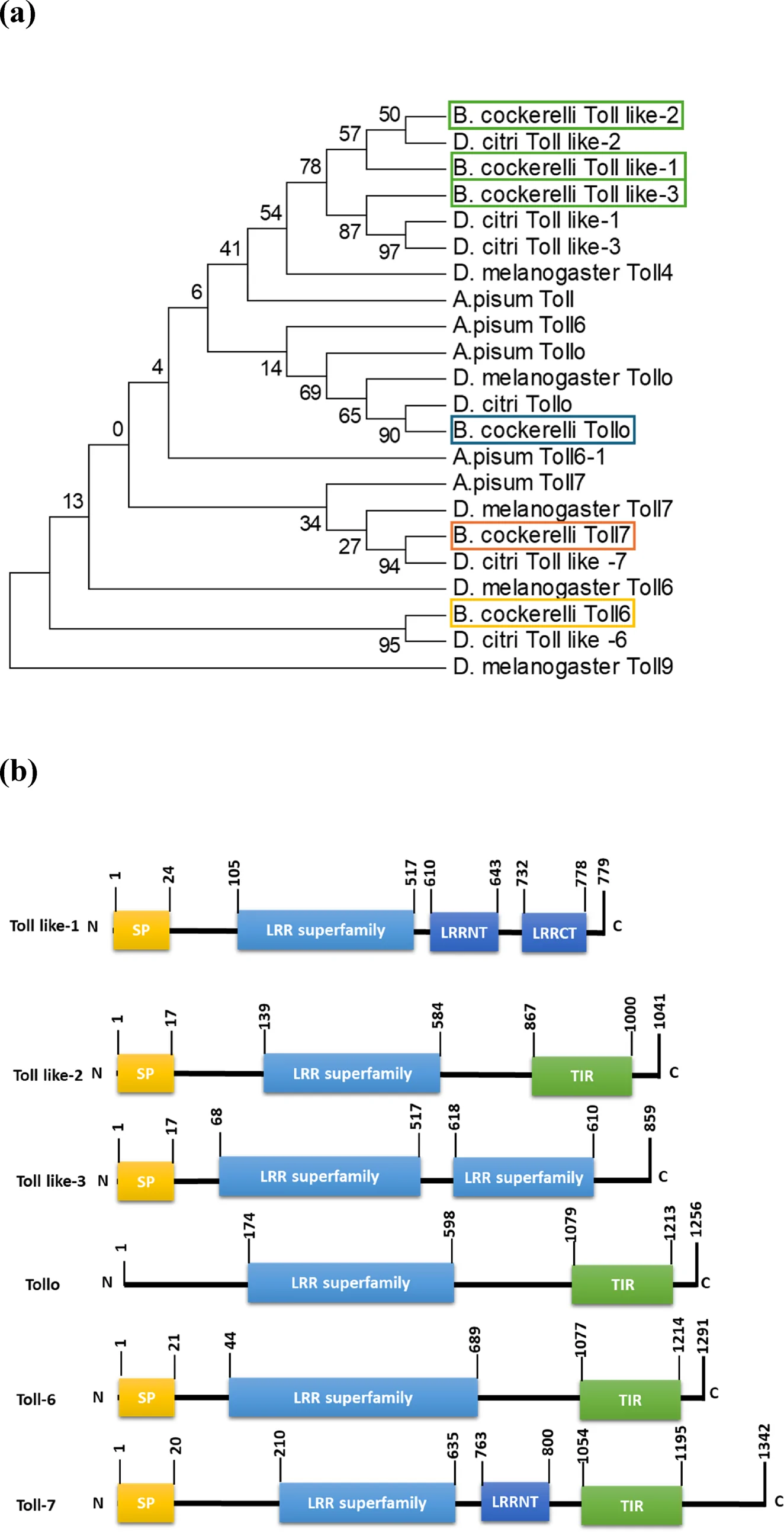
Analysis of putative *B. cockerelli* Toll genes. (**a**) Phylogenetic analysis of six Toll proteins. The analysis was performed using the Maximum Likelihood method in MEGA6. Bootstrapping values were obtained with 1000 repetitions to support branching and clustering. Insect species include *Acyrthosiphon pisum*, *Drosophila melanogaster*, and *Diaphorina citri*. (**b**) Domain prediction of Toll proteins using Prosite (https://prosite.expasy.org/) and InterPro (https://www.ebi.ac.uk/interpro/). ‘SP’ stands for signal peptide

Six Toll receptor genes were identified in the *B. cockerelli* genome sequence. Phylogenetic analysis of the Toll receptor proteins in the evolutionary relationship between the potato psyllid, *D. citri*, *A. pisum*, and *D. melanogaster* revealed that the six Toll showed high similarity to *D. citri*’s proteins and were named Toll like-1 (PX869299), Toll like-2 (PX869300), Toll like-3 (PX869303), Toll-6 (PX869302), Toll-7 (PX869301), and Tollo (PX869304) (Fig. 1a). Conserved domain analysis confirmed that the identified candidates were members of the Toll receptor family (Fig. 1b). All candidate receptors encoded the characteristic Toll receptor domain architecture, including an extracellular leucine-rich repeat (LRR) domain and an intracellular Toll/interleukin-1 receptor (TIR) domain, except for Toll like-1 and Toll like-3 which lacked this latter domain. In addition, five candidates encoded a signal peptide at the N-terminus, whereas Tollo lacked a predicted signal peptide.

**Fig. 2:**
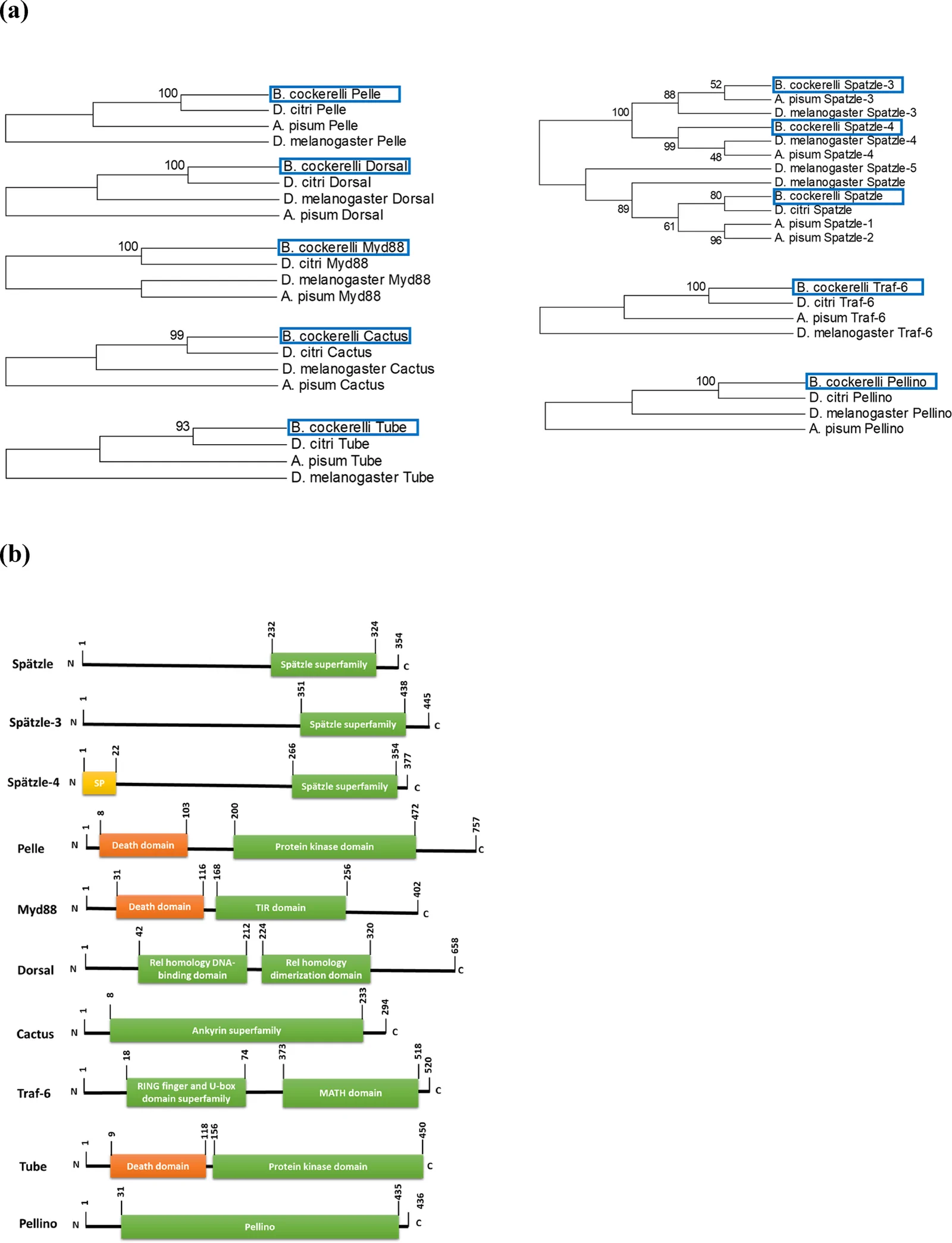
Identification and bioinformatics analysis of Toll pathway-related genes. (**a**) Phylogenetic analysis of Spätzle, Myd88, Tube, Dorsal, Cactus, Traf-6, Pelle and Pellino proteins. The analysis was performed using the Maximum Likelihood method in MEGA6. Bootstrapping values were obtained with 1000 repetitions to support branching and clustering. Insect species include *Acyrthosiphon pisum*, *Drosophila melanogaster*, and *Diaphorina citri.* (**b**) Domain analysis of Spätzle, Myd88, Tube, Dorsal, Cactus, Traf-6, Pelle, and Pellino proteins. ‘SP’ stands for signal peptide. Domains were predicted using Prosite (https://prosite.expasy.org/) and InterPro (https://www.ebi.ac.uk/interpro/)

A total of ten genes associated with the Toll signaling pathway were identified. Three potato psyllid Spätzle genes were identified and named *Spätzle* (PX876874), *Spätzle-3* (PX876871), and *Spätzle-4* (PX876872) based on their similarity to *A. pisum*, *D. melanogaster* and/or *D. citri* proteins. The other genes included one putative *Pelle* (PX876865), *MyD88* (PX876866), *Dorsal* (PX876867), *Cactus* (PX876868), *Traf-6* (PX876869), *Tube* (PX876873), and *Pellino* (PX876870). The latter showed the highest similarity to *D. citri* proteins. (Fig. 2a).

Conserved domain analysis demonstrated that all Spätzle proteins possessed a C-terminal Spätzle superfamily domain and Spätzle-4 also encoded an N-terminal signal peptide. Pelle and Tube each contained a death domain and a protein kinase domain. The adaptor protein MyD88 encoded both a death domain and a TIR domain. The transcription factor Dorsal contained two Rel homology regions, consisting of a DNA-binding domain and a dimerization domain. Additionally, Cactus possessed ankyrin superfamily domains, and Traf-6 encoded both a RING finger/U-box domain and a MATH (Meprin and TRAF homology domain) and Pellino contained a Pellino domain. (Fig. 2b).

### Bactericera cockerelli toll receptors were up-regulated upon fungal challenge

**Fig. 3:**
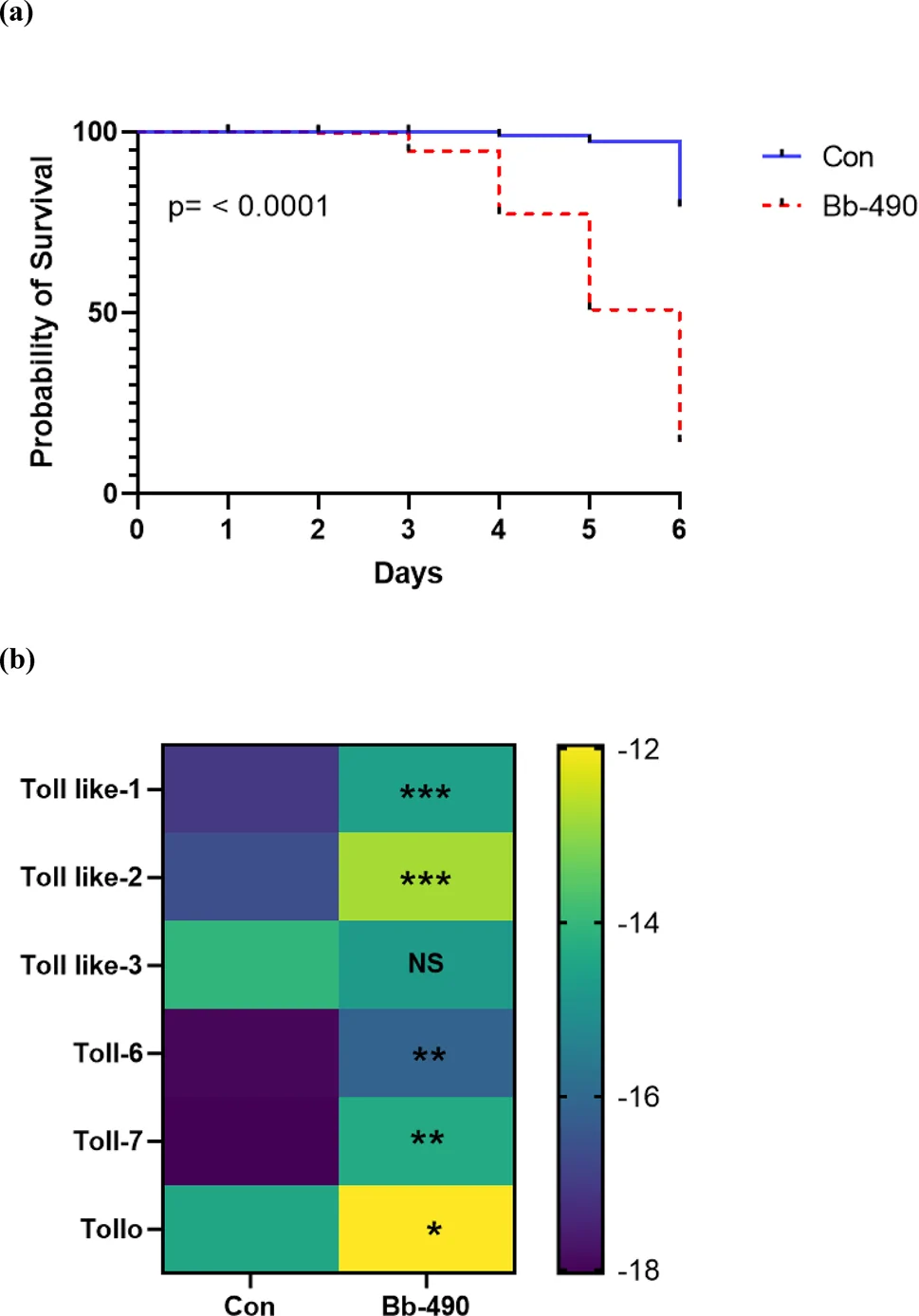
Toxicity effect of Bb-490 against potato psyllid nymphs. (**a**) Mortality of Lso-free nymphs following feeding on fungus- and control-treated leaves. Data was recorded every 24 h for six days. (**b**) Heat map of the relative gene expression of *Toll like-1*, *Toll like-2*, *Toll like-3*, *Toll-6*, *Toll-7*, and *Tollo* following each treatment. Each row represents a specific gene, and each column corresponds to the control and fungus treatment. The color scale represents minus ΔCT values, with dark purple indicating low expression and bright yellow indicating high expression. Statistical analysis was performed using Student’s *t*-test to compare the expression levels of each gene between control and treatment: *p* < 0.01 (**), *p* < 0.001 (***), and *p* < 0.0001 (****); NS indicates not significant. Con = Control and Bb-490 = fungus treatment

To evaluate the potential involvement of the identified candidates in the immune response, first we determined the insecticidal activity *of Bb-490* strains against third-instar nymphs (Fig. 3a). Higher mortality was observed for nymphs placed on leaves treated with *Bb-490* compared to the control (leaves dipped in the solution without the fungus). Specifically, high mortality was observed after 3 days of treatment, and by the 6th day, on average 82% of the nymphs had died. Lower mortality was observed in the control treatment, whereby at the end of the bioassay, on average, 80% of the nymphs remained alive. Then, the effect of *Bb-490* on the expression of Toll genes was assessed in *B. cockerelli* L3 nymphs. Among the six Toll genes examined, five were up-regulated at 48 h post-challenge (Fig. 3b).

### Lso haplotypes regulated the toll pathway in a haplotype-specific manner

To determine the transcriptional regulation of the identified genes following Lso infection, we first evaluated Lso titers following different AAPs (Fig. S1). There was a significant effect of the haplotype (F(1, 48) = 21.04, *p* = 0.0001) and of the AAP (F(5, 48) = 67.45, *p* = 0.0001) on the number of Lso copies in the psyllid. However, the interaction of Lso haplotype and AAP on the number of Lso copies was not significant (F(5, 48) = 1.86, *p* = 0.1188). Although LsoA titer increased more slowly, it was similar to LsoB titer after 5 days AAP.

**Fig. 4:**
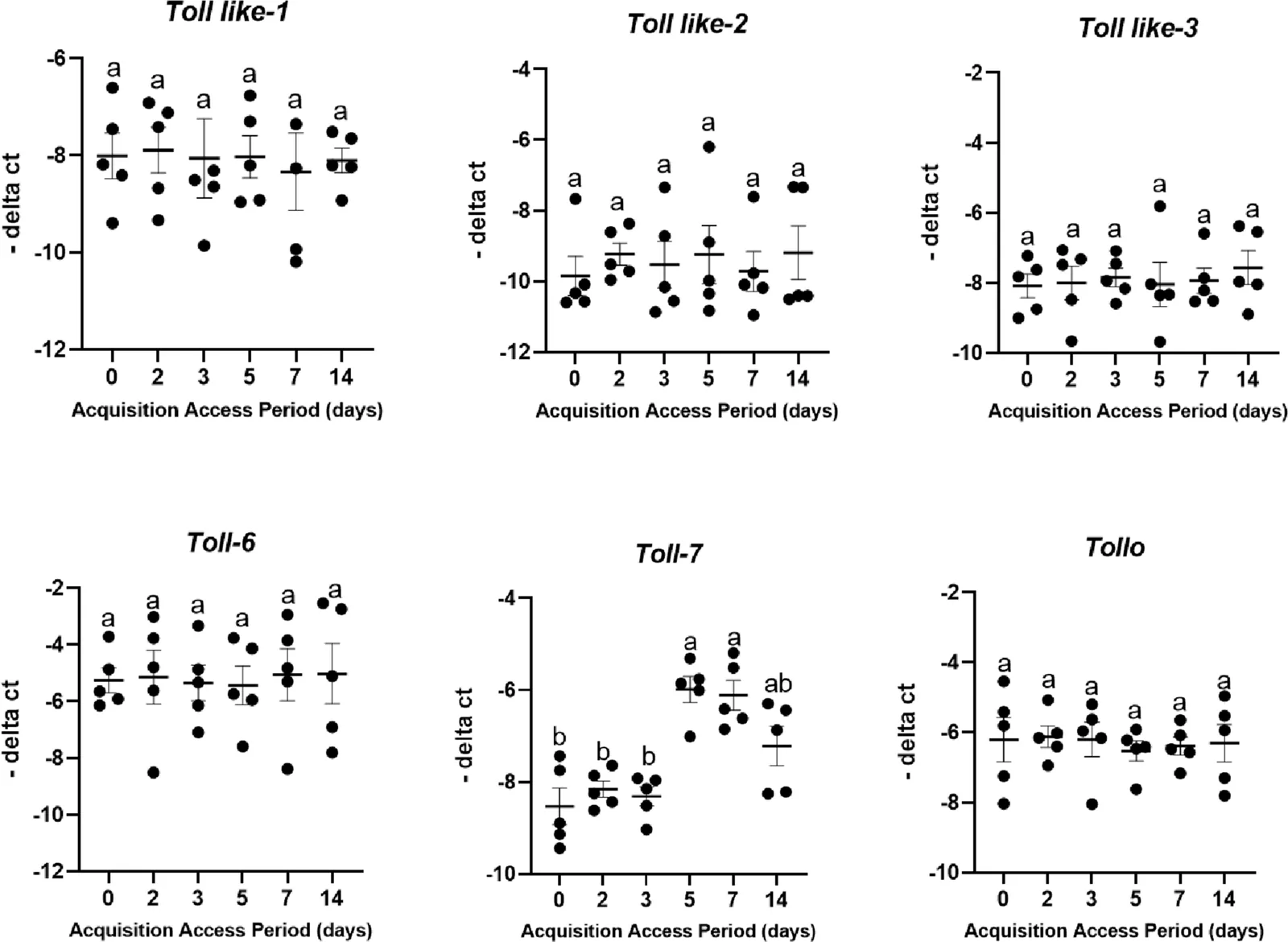
Regulation of Toll genes in psyllid adults upon LsoA infection. Data represent means ± SE of five independent experiments. Different letters indicate statistical differences at *p* < 0.05 using one-way ANOVA with Tukey’s *post hoc* test. 0 indicates Lso-free insects; 2, 3, 5, 7 and 14 represent the different time points in days of LsoA infection

**Fig. 5:**
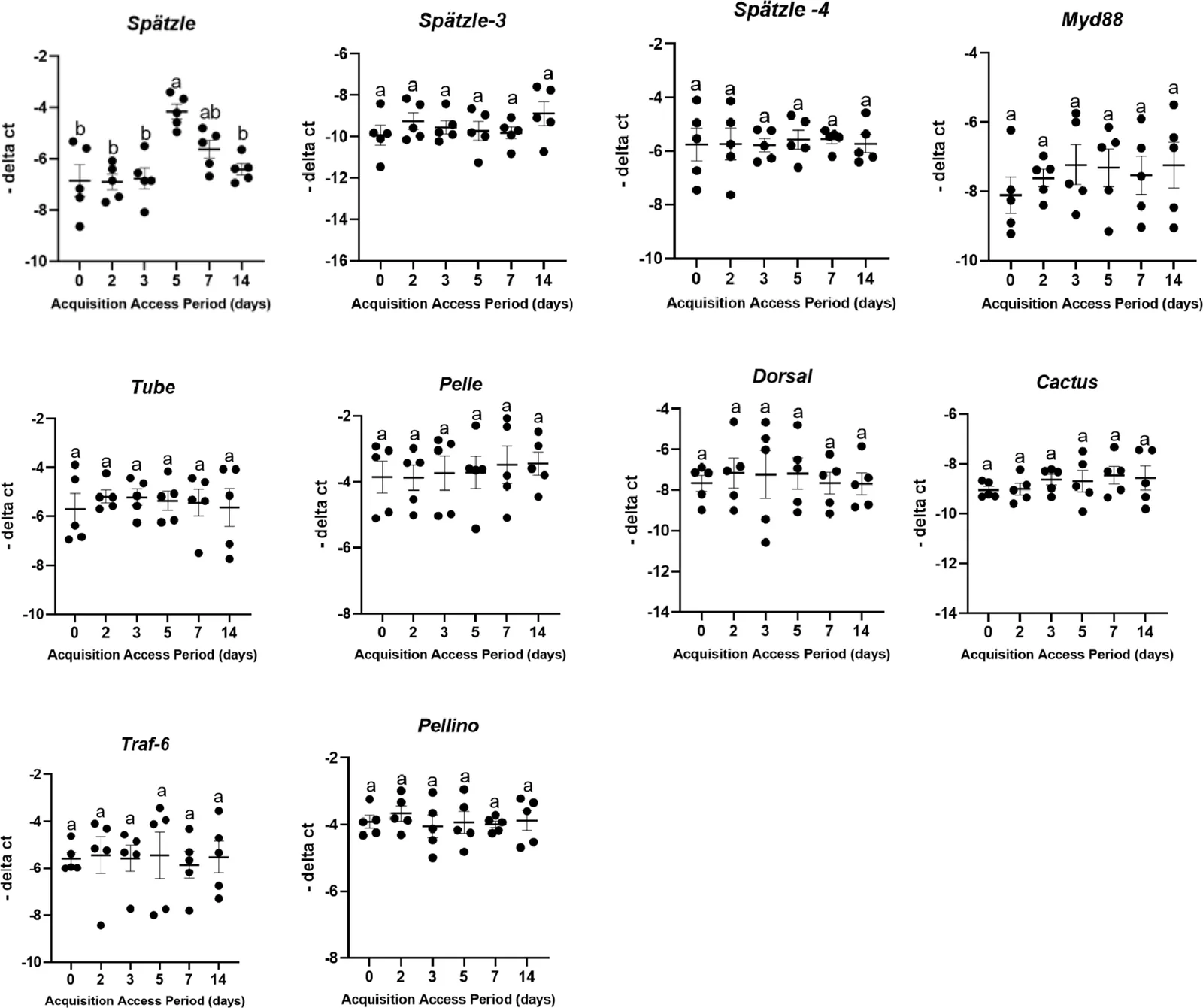
Regulation of *Spätzle*, *Spätzle-3*, *Spätzle-4*, *Myd88*, *Tube*, *Pelle*, *Dorsal*, *Cactus*, *Traf-6* and *Pellino* upon LsoA infection. Different letters indicate statistical differences among means at *p* < 0.05 (Tukey’s *post hoc*). 0 indicates Lso-free insects; 2, 3, 5, 7 and 14 represent the different time points in days of LsoA infection

The expression profile of the Toll genes was determined in adults following 2, 3, 5, 7, and 14 days of AAP on LsoA and LsoB-infected plants. In response to LsoA acquisition, only *Toll-7* was up-regulated: its expression increased at 5 and 7 days post-acquisition (Fig. 4). Similarly, *Spätzle* was significantly up-regulated by LsoA at 5 days AAP (Fig. 5).

**Fig. 6:**
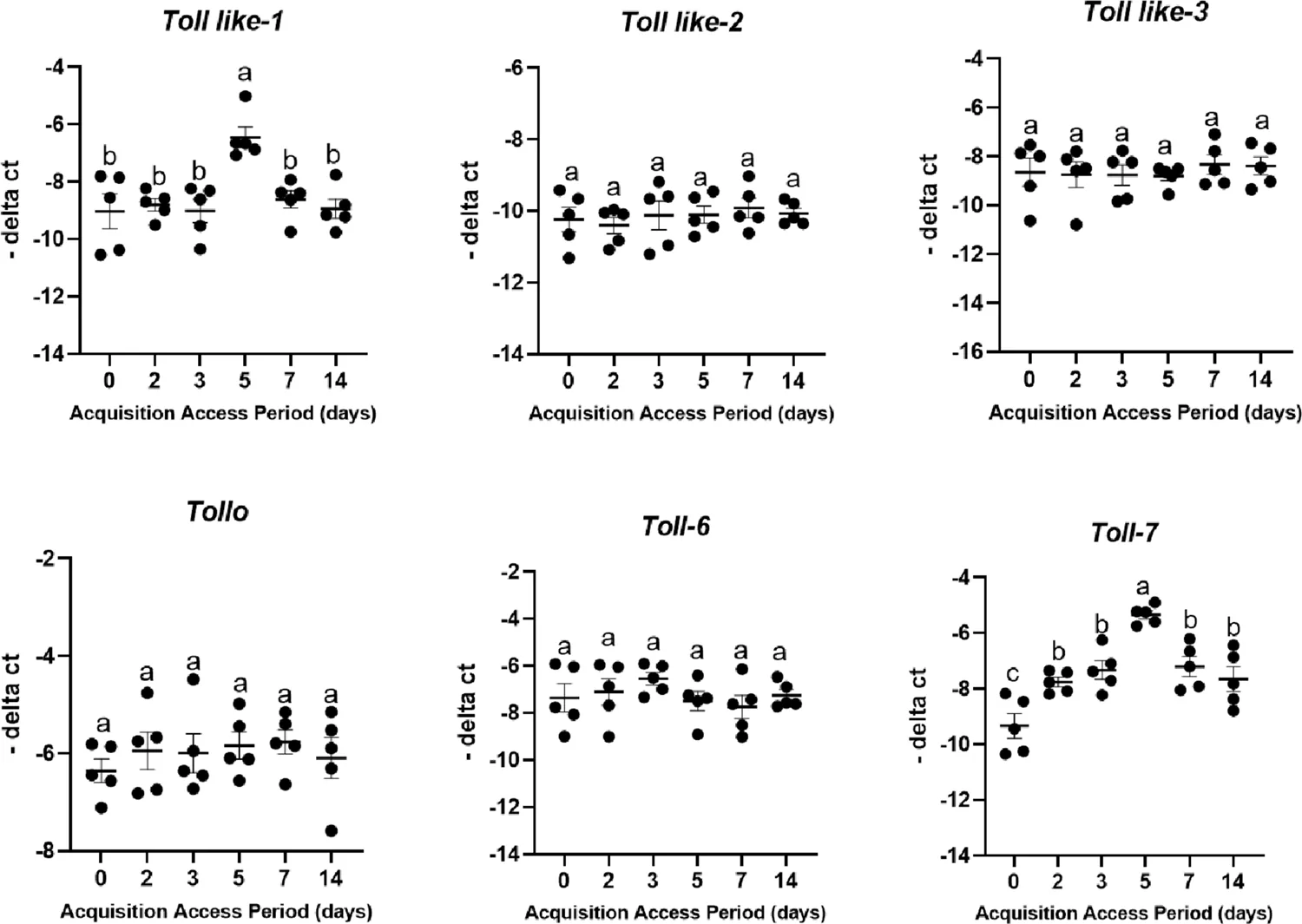
Regulation of the Toll genes in psyllid adults upon LsoB infection. Data represent means ± SE of five independent biological replicates. Different letters indicate statistical differences among means at *p* < 0.05 (Tukey’s *post hoc*). 0 indicates Lso-free insects; 2, 3, 5, 7 and 14 represent the different time points in days of LsoB infection

**Fig. 7:**
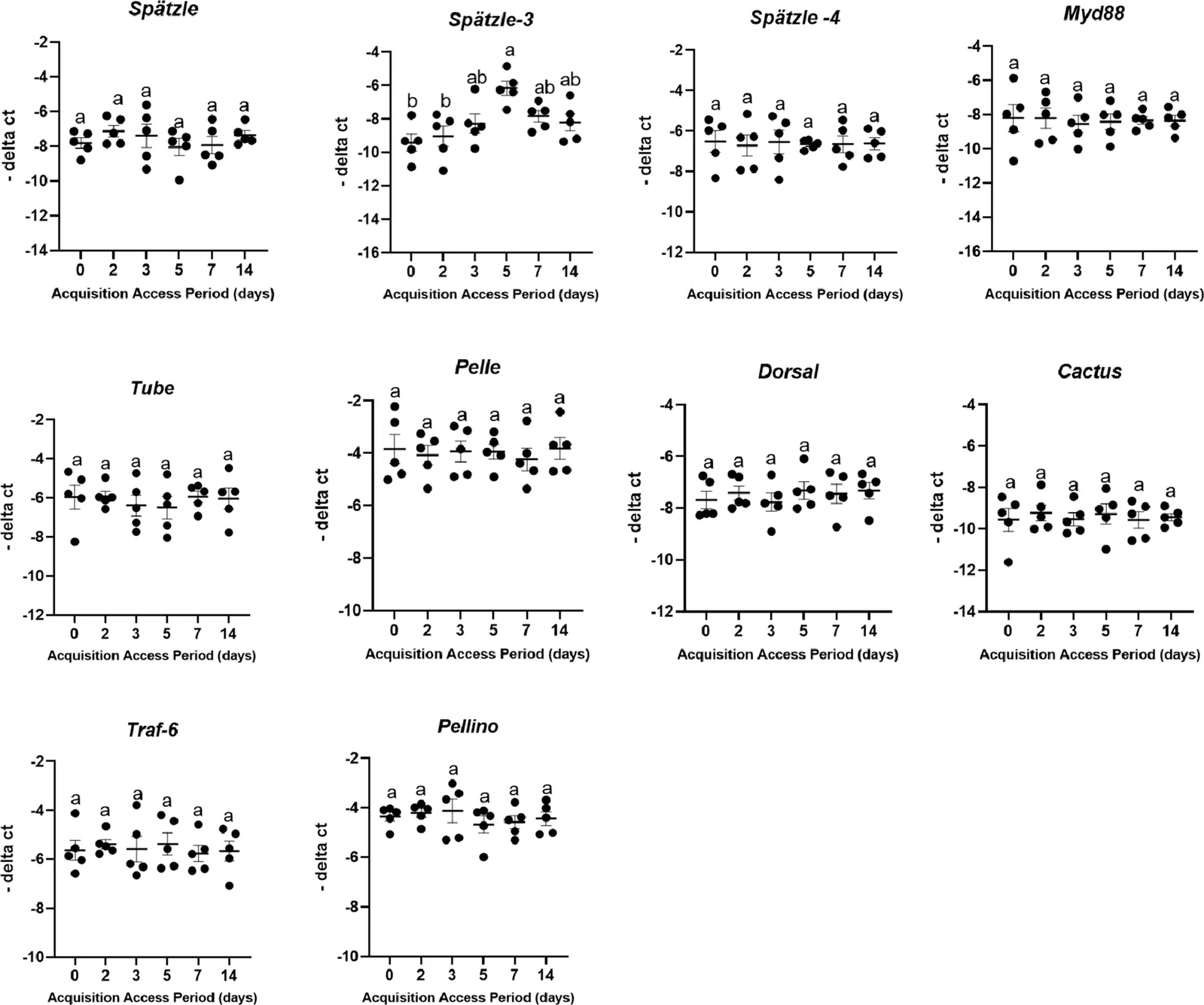
Expression profile of *Spätzle*, *Spätzle-3*, *Spätzle-4*, *Myd88*, *Tube*, *Pelle*, *Dorsal*, *Cactus*, *Traf-6* and *Pellino* upon LsoB infection. Different letters indicate statistical differences among means at *p* < 0.05 (Tukey’s *post hoc*). 0 indicates Lso-free insects; 2, 3, 5, 7 and 14 represent the different time points in days of LsoB infection

However, *Toll like-1*, and *Toll-7*, were up-regulated at 5-days AAP on insects that acquired LsoB (Fig. 6). Similarly, *Spätzle-3* was significantly up-regulated by LsoB at 5 days AAP (Fig. 7). The other genes, including *Spätzle*,* Spätzle-4*, *Myd88*, *Tube*, *Pelle*, *Dorsal*, *Cactus*, *Traf-6*, and *Pellino* showed no significant change in expression under either infection condition. These dynamic expressions of Toll pathway-related genes demonstrated the potentially active and complex involvement of the Toll signaling pathway in response to infection by LsoA and LsoB.

### The toll genes were not regulated by the persistent LsoA or LsoB infection

**Fig. 8:**
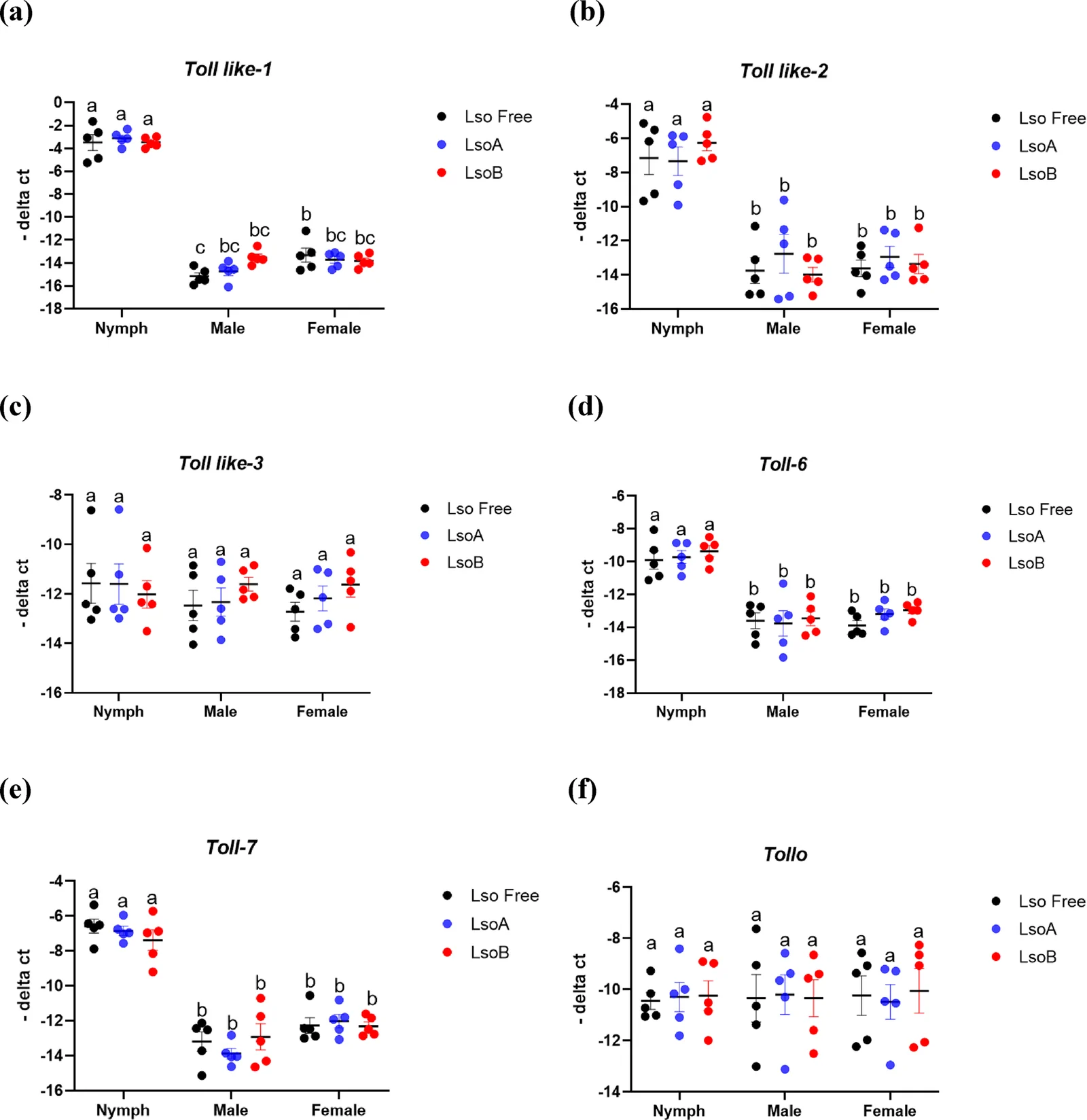
The mRNA expression profile of (**a**) *Toll like-1*, (**b**) *Toll like-2*, **(c**) *Toll like-3*, (**d**) *Toll-6*, (**e**) *Toll-7*, and **(f**) *Tollo* genes in response to LsoA and LsoB persistent infection in 5th instar nymphs, adult males, and adult females. Each treatment was replicated five times with independent biological sample preparations. Different letters indicate significant differences among means at *p* < 0.05

A two-way ANOVA was conducted to assess the effects of developmental stage (nymph, adult female, adult male) and treatment (Lso-free, LsoA-infected, LsoB-infected) on the expression of various Toll genes in the psyllid. The analysis revealed a significant effect of developmental stage on the expression levels of *Toll like-1* (F (2, 36) = 732.91, *p* < 0.0001), *Toll like-2* (F (2, 36) = 79.17, *p* < 0.0001), *Toll-6* (F (2, 36) = 71.37, *p* < 0.0001), and *Toll-7* (F(2, 36) = 161.93, *p* < 0.0001). However, no significant effect of developmental stage was found on the expression levels of *Toll like-3*, (2, 36) = 0.5375, *p* = 0.5888)and *Tollo* (F (2, 36) = 0.0064, *p* = 0.9936). Furthermore, neither the infection nor the interaction between developmental stage and infection (Fig. 8) had a statistically significant effect on the expression of the genes (*p* > 0.05).

## Discussion

Three primary signaling pathways play roles in insect innate immunity in response to pathogens: Toll, IMD, and JAK/STAT [14]. In *D. melanogaster*, viruses, fungi, and Gram-positive bacteria activate the Toll pathway [34, 35], whereas Gram-negative bacteria activate the IMD pathway [36]. This canonical model was found to be inconsistent in other insect species, including dipterans [37]. In hemipteran insects lacking a complete IMD pathway, the Toll pathway is also activated in response to Gram-negative bacteria in *Myzus persicae* [38]. The potato psyllid encodes the Toll pathway but lacks a complete IMD pathway [39], suggesting that this insect might have a reduced immune response to Gram-negative bacteria such as Lso. However, it is also possible that the potato psyllid employs other defense mechanisms, such as endosymbiont-mediated or cellular immune responses, as suggested for *D. citri* [40]. This reduction may contribute to the psyllid’s susceptibility to infection by Lso.

The Toll pathway plays a crucial role in mediating innate immune responses and triggering the production of antimicrobial peptides (AMPs) in certain insect species. Here, six Toll receptor genes were identified in the potato psyllid, which is fewer compared to other insects; for instance, seven Toll-related genes were identified in *Acyrthosiphon pisum* and 14 in *Bombyx mori* [21, 41], however, six were identified in *D.* citri [40]. Sequence analyses indicated that conserved domains of the Toll family were found in the predicted Toll amino acid sequences, and a similar conservation has also been found in Toll sequences in *B. mori*,* D. melanogaster*,* and D. citri* [42–44]. Among the six Toll receptors identified, four possessed both the extracellular leucine-rich repeat (LRR) domain and the intracellular Toll/interleukin-1 receptor (TIR) domain. The LRR domain mediates ligand recognition, while the TIR domain is essential for recruiting adaptor proteins such as MyD88, thereby initiating downstream NF-κB–dependent immune signaling.

In contrast, two Toll receptors contained only the LRR domain and lacked the TIR domain, similar to observations in *D. citri*. We speculate that the absence of the TIR domain means that these receptors are unlikely to independently transduce intracellular signals through the classical Toll pathway. Instead, these LRR-only receptors may function primarily as ligand-binding receptors that relay signals to other Toll receptors, thereby modulating downstream immune activation. Additionally, Tollo lacks a signal peptide compared to other Toll receptors, a similar feature also reported in *D. citri*, which may influence its cellular localization and function.

Toll genes are usually induced in response to fungus. Therefore, to evaluate if the identified Toll genes could be involved in fungal defense, we used the entomopathogenic fungus, *B. bassiana*, which is registered to control insects all over the world. We determined that Bb-490 possesses high toxicity against the 3rd instar nymphs of the potato psyllid, causing significant mortality. Furthermore, five Toll genes evaluated were significantly up-regulated upon fungal infection, which confirmed their potential involvement in the psyllid response to fungal infection.

The Toll pathway was originally thought to be mainly triggered by Gram-positive bacteria and fungi, regulating the production of most antifungal peptides. Nonetheless, Gram-negative bacterial infections have been shown to activate the Toll pathway in *Bombyx mori* [45]. In this study, the expression of the Toll genes varied in response to Lso infection: two Toll receptor genes and *Spätzle-3* were up-regulated by LsoB at day 5 of the AAP, while only *Toll-7* and *Spätzle* were up-regulated after 5 days of AAP by LsoA. These results indicate the differential involvement of these Toll genes in response to the specific Lso haplotypes. Indeed, different transcriptomic responses are induced in response to LsoA and LsoB [46], with more differentially expressed genes following LsoB infection. Furthermore, while LsoB accumulated more rapidly than LsoA in psyllids, it cannot be excluded that the expression of these genes might be influenced by the amount of Lso present in the insect. However, changes in gene expression were mainly observed at 5 days of AAP, a time point after which similar LsoA and LsoB titers were measured in psyllids. Therefore, these results suggest that gene expression is linked to the time of infection, not the bacterial titer.

The differential expression of the *Spätzle* genes suggests that distinct *Spätzle* may play specialized roles in activating the Toll signaling pathway in response to different Lso haplotypes. Similar findings have been reported in other insects, where Toll family members can bind multiple Spätzle proteins, to activate Toll-immune signaling in *Drosophila* [47]. However, other downstream components of the Toll pathway, including *Myd88*,* Tube*,* Pelle*,* Dorsal*,* Cactus*, *Traf-6* and *Pellino* did not show significant transcriptional changes following LsoA or LsoB infection. This indicates that these genes are transcriptionally stable in response to Lso infection under the conditions examined.

Additionally, the analysis of the expression of Toll genes in response to the persistent Lso infection in nymphs, adult males and females showed that none of the genes were regulated in psyllids infected with either LsoA or LsoB. This lack of regulation of the Toll genes during the persistent infection is notable, as it might imply that Lso is able to manipulate the psyllid Toll pathway. Furthermore, these results validate the need to investigate the insect responses upon infection and not just during the persistent infection. So, we hypothesized that activation of the Toll pathway could occur in the psyllid upon LsoA and LsoB infection.

While Toll genes were up-regulated in response to LsoB and LsoA, this up-regulation was not a sufficient defense because both LsoA and LsoB can be successfully transmitted by potato psyllids. A plausible explanation is that, although the Toll pathway is activated in response to the pathogen, this defense mechanism may be either inadequate or not sufficiently widespread to effectively halt Lso infection. A similar scenario occurs in response to the fungal challenge, which up-regulated five Toll genes, but resulted in high psyllid mortality.

## Supplementary Information

Below is the link to the electronic supplementary material.

Supplementary Material 1

## Data Availability

All data were included in the manuscript.
