# Supplementary material for: Identification and gene expression profiling of toll genes in the potato psyllid (Bactericera cockerelli) in response to ‘Candidatus liberibacter solanacearum’
Source: Mol Biol Rep. 2026 Sep 22;53(1):1605. doi: 10.1007/s11033-026-12777-9 (PMC13597544; doi:10.1007/s11033-026-12777-9)
Supplement: Supplementary file 1 [file 11033_2026_12777_MOESM1_ESM.docx]

**Fig. S1**

**Figure caption**

**Fig. S1:** Quantification of LsoA and LsoB titers in psyllids following acquisition. Titers of LsoA (blue) and LsoB (red) were measured after a 0-, 2-, 3-, 5-, 7- and 14-day acquisition access period (AAP). Data presented are mean ± standard error (SE) from five independent biological replicates. Different letters indicate statistical differences at *P* < 0.05 using two-way ANOVA with Tukey's *post hoc* test.

Table S1. Primers used in this study

| **Genes** | **Orientation** | **Sequence** | **Primer efficiency (%)** | **Uses** |
| --- | --- | --- | --- | --- |
| **Toll like-1** | Forward | CCAAGAAGTGCCTGTGGACT | 96.7 | qPCR |
|  | Reverse | TTGAAACAAATCTTTAGGAA |  |  |
| **Toll like-2** | Forward | GCTTTTGAACCGCTCAAGCA | 95.5 | qPCR |
|  | Reverse | GGGTGCATGATTCTAGTGGA |  |  |
| **Toll like-3** | Forward | CCCTAATGGCACTACCTGGC | 95.7 | qPCR |
|  | Reverse | AACATCGGGTGGAACTGTCC |  |  |
| **Toll-6** | Reverse | GCTTCCTGGTCTTTCGTCCA | 95.7 | qPCR |
|  | Reverse | ATTGGCCACAAAATCTGAAA |  |  |
| **Toll-7** | Forward | TCTCGCTAGCTCTGTGTCCT | 97.3 | qPCR |
|  | Reverse | CACATCCACTGGAAGATCCA |  |  |
| **Tollo** | Forward | GCTCAGGGTTTTGCAACTCG | 95.7 | qPCR |
|  | Reverse | GGGTTAGCCGTGAGAGTGAC |  |  |
| **Spätzle** | Forward | CAGAAACACAAGGCGTGCAA | 96.8 | qPCR |
|  | Reverse | CCACCAGCTTGCGGTACATA |  |  |
| **Spätzle-3** | Forward | CTAGTCCGTCGGGTTATGGC | 95.8 | qPCR |
|  | Reverse | GGTAGCTCTCGGGTGCTTTT |  |  |
| **Spätzle-4** | Forward | TGTGACCTCCGCACTCAATC | 97.5 | qPCR |
|  | Reverse | TTCATGAGGCCGTGGTTCTC |  |  |
| **Pelle** | Forward | TGCCAGCGAGAACAATCCAT | 95.3 | qPCR |
|  | Reverse | ACTTGCCAGACTCAAAACTGA |  |  |
| **MyD88** | Forward | GAACCGACTACTGCACCCAT | 93.6 | qPCR |
|  | Reverse | GTTTCCGCTGCAGCAGAAGA |  |  |
| **Dorsal** | Forward | TTCATCGACCGGAACAGTGG | 94.9 | qPCR |
|  | Reverse | GACGAGGGGGCGGGGTCTG |  |  |
| **Cactus** | Forward | GTCAGTGGCGGAGAGTAACC | 94.3 | qPCR |
|  | Reverse | CCGCGGCTCGTACTTTAACC |  |  |
| **Traf-6** | Forward | CCCGTGAGGATCCCCAGTAT | 96.2 | qPCR |
|  | Reverse | ATCTGTCCAACAAACGGCCA |  |  |
| **Tube** | Forward | AATGCAAACACGCCAACCTC |  | qPCR |
|  | Reverse | CAGAGATCCGTTGGGCATGT | 96.4 |  |
| **Pellino** | Forward | GGCCAAATCACCGACGAAAG | 96.9 | qPCR |
|  | Reverse | GGATGACCAGGGTGTTGAGG |  |  |
